# Supplementary material for: Mothers with Cancer: An Intersectional Mixed-Methods Study Investigating Role Demands and Perceived Coping Abilities
Source: Cancers (Basel). 2023 Mar 22;15(6):1915. doi: 10.3390/cancers15061915 (PMC10047435; doi:10.3390/cancers15061915)

Supplement Figure S1. Focus group session schedule handout.

Agenda

1. Introduction
2. Presentation on the themes developed from the interviews
3. Discussion on the accuracy and comprehensiveness of the themes
4. Presentation of the model
5. Discussion on the model and how it should be applied
6. Following up on some questions from the original interviews
7. Closing

Total time: 90 minutes

Themes

The following themes were developed after compiling all interviews and looking for recurring trends and experiences surrounding being a mother with cancer.

1. **Navigating roles and role conflict**
    a. Parental role demands were prioritized, followed by cancer, relationship (e.g., friends, spouses, etc.), and work (formal and informal)
    b. Cancer patient role demands
        i. Physical and cognitive challenges (e.g., mobility limitations post-surgery, tiredness, brain fog, etc.)
        ii. Attending appointments
        iii. Getting sufficient rest
    c. Parental role demands
        i. Discussing cancer diagnosis with children was helpful
        ii. Older (jr. high, high school)
            1. Take on more household demands
            2. Sensitive to mother's cancer
            3. Typical "teenage problems" persist (avoiding chores, emotional volatility, skipping school, sneaking out, mental health challenges)
        iii. Younger (toddler, elementary)
            1. Expectations of mother doesn't change
            2. Helps mothers feel normal
    d. Relationship role demands
        i. Mothers take on increased emotional labour demands in each role (e.g., having to support loved ones as they cope with the cancer diagnosis)

        ii. Intimate partner (partners can be a source of support but may struggle to provide sufficient emotional support, maintaining a sexual relationship can be challenging)
        iii. Friendships (desire for relationship reciprocity)
        iv. Daughter (transitioning back into the “cared for” role)
        v. Caregiver (child/elder care demands remained on the mother)
    e. Work role demands (formal and informal)
        i. Shift in pragmatic tasks to a partner
        ii. Woman still took on most of the organizational role demands
        iii. Accepting help from the community eased practical demands (e.g., neighbours or church brought food)
        iv. Previous strong communication and egalitarianism was protective for relationship stress (partners carried their demands forward and were more able to take on others)

2. **Motivation and strategies used to cope with role demands**
    a. Communication
    b. Receiving social support
    c. Mothers had to learn to ask for/accept help
    d. Self-care
    e. Scheduling parental role demands around cancer demands (appointments/sleep) to avoid role conflict
3. **Sense of self as it relates to the capacity to fulfill role demands (identity)**
    a. Loss of sense of self when separated from roles
    b. Internal pressure to meet role demands/role overload
    c. Self-esteem tied to being a helper
    d. Career being lost and fear of returning
    e. Ability to uphold womanhood (makeup, hair, body weight, etc.) impacted sense of self
4. **Perception of quality of life**
    a. Seeking balance
    b. Perspective change (e.g., being more present focused, shifting away from material things, focus on nature, etc.)
    c. More aware of energy availability
    d. Wanting to have a sense of normalcy

Model

The following model was developed by considering the experiences and perspectives expressed in the interviews and questionnaires. The intended purpose of this model is to be used as an educational resource for mothers with cancer and their families, clinicians, and researchers.

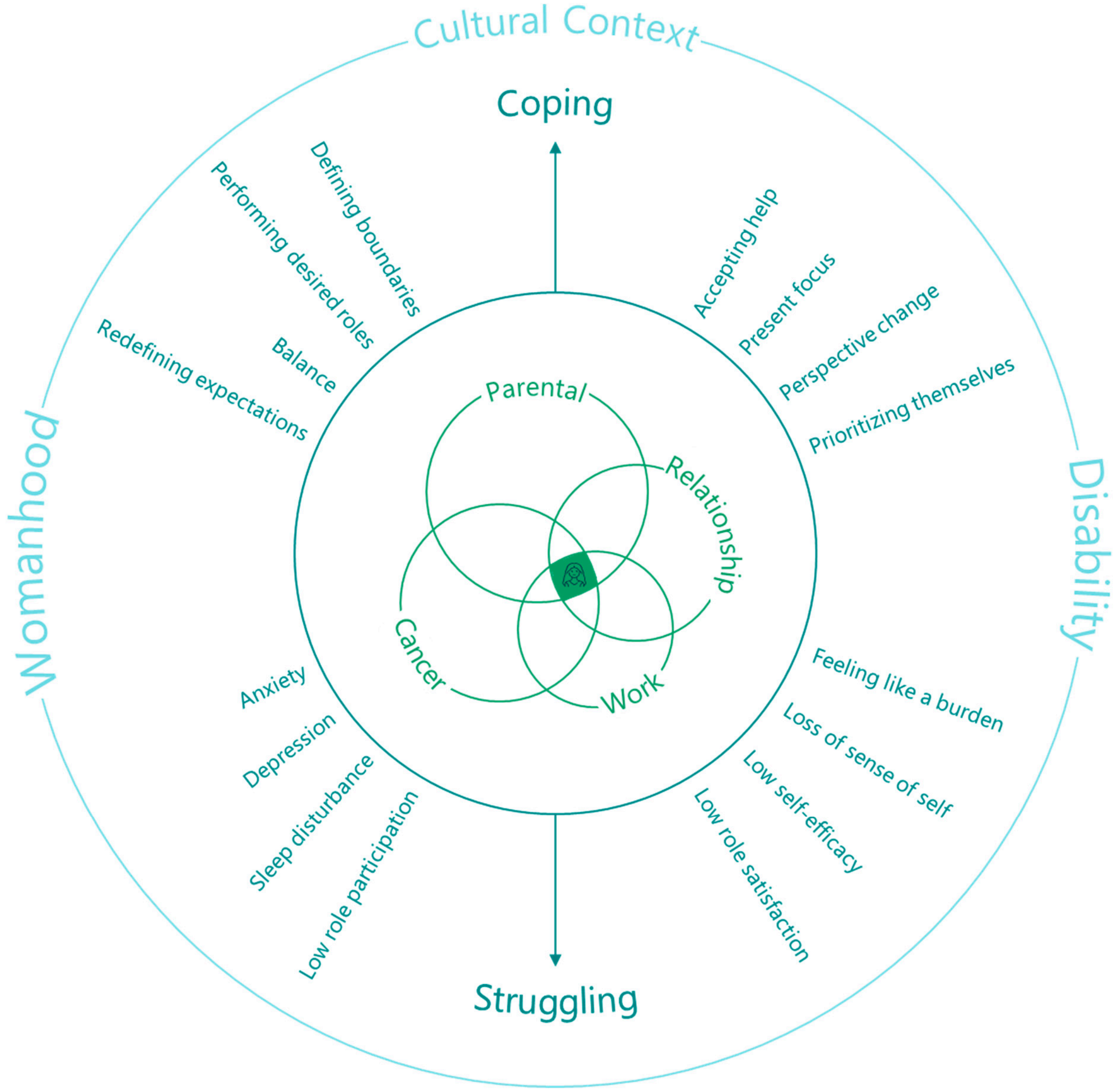

Supplement: Supplementary file 1 [file cancers-15-01915-s001.zip › Supplement Figure S1_MwC.pdf]
