# Supplementary material for: Mothers with Cancer: An Intersectional Mixed-Methods Study Investigating Role Demands and Perceived Coping Abilities
Source: Cancers (Basel). 2023 Mar 22;15(6):1915. doi: 10.3390/cancers15061915 (PMC10047435; doi:10.3390/cancers15061915)
Supplement: Supplementary file 1 [file cancers-15-01915-s001.zip › Supplement Table S1_MwC.pdf]

Supplement Table S1. Semi-structured interview guide based on study aims.

Study aim		Guiding interview questions
(i)	Determine what roles mothers with cancer assume and how cancer impacts role functioning	What roles do you experience and what are the accompanying role demands?
		Which roles do you feel are particularly demanding? How do you prioritize your role demands?
		How do you feel cancer has impacted your ability to meet your role demands?
(ii)	Identify how mothers' perceived ability to cope with role demands impacts psychosocial functioning and QoL	What strategies have you used to cope when you are struggling?
		Do you practice self care? If so, what activities do you do and how often?
		What motivates you to meet your role demands when you are struggling?
		What goes into making a good QoL? How would you describe your current QoL? How do your role demands fit into the ways in which cancer has affected your QoL?
(iii)	Explore how the social context surrounding disability and womanhood impact role functioning	What expectations do you feel the people in your life have for you in terms of your role demands?
		How do you feel people in your life treat you if you are not able to meet your role demands?
		How do you feel people treat you when you go above and beyond your role demands?
		Do you ever feel pressure to "do-it-all" and does this impact your sense-of-self?

Note. Participants will be given the definition of role demands at the beginning of interviews: role demands are similar to role stressors, in that role demands are the requirements associated with the roles you occupy. One person can occupy many roles, such as being a mother, an employee, a wife, or in cancer treatment, and all of these roles interact and influence each other.
