# Supplementary material for: Mothers with Cancer: An Intersectional Mixed-Methods Study Investigating Role Demands and Perceived Coping Abilities
Source: Cancers (Basel). 2023 Mar 22;15(6):1915. doi: 10.3390/cancers15061915 (PMC10047435; doi:10.3390/cancers15061915)
Supplement: Supplementary file 1 [file cancers-15-01915-s001.zip › Supplement Table S2_MwC.pdf]

Supplement Table S2. Overview of focus group transcript analysis.

Phase	Coding method	Performed by
1. Researchers familiarizing themselves with the data		A.S., volunteer 1, volunteer 2
2. Generating initial codes	Inductive approach	A.S., volunteer 1, volunteer 2
Iterative process	Consensus-based codebook	A.S., volunteer 1, volunteer 2
Review	Consensus-based codebook	A.S., volunteer 1, volunteer 2, S.B.
Data saturation	Final codebook	A.S., volunteer 1, volunteer 2
3. Searching for themes	Consensus approach based on emergent themes	A.S., volunteer 1, volunteer 2
4. Reviewing themes	Consensus approach based on previously defined interview themes	A.S., volunteer 1, volunteer 2, S.B.
5. Defining themes	Consensus approach based on emergent themes and literature	A.S., volunteer 1, volunteer 2, S.B.,
6. Producing a report		A.S., volunteer 1, volunteer 2, S.B., J.D.

Note. Coding was conducted according to a-priori content using a liberal
